# Supplementary material for: Identification of a quasi-liquid phase at solid–liquid interface
Source: Nat Commun. 2022 Jun 23;13:3601. doi: 10.1038/s41467-022-31075-z (PMC9226024; doi:10.1038/s41467-022-31075-z)
Supplement: Supplementary file 1 — Supplementary Information [file 41467_2022_31075_MOESM1_ESM.docx]

**Supplementary Information**

*for*

**Identification of a Quasi-Liquid Phase at Solid-Liquid Interface**

Xinxing Peng^1,2,†,#^, Fu-Chun Zhu^1,†^, You-Hong Jiang^1,†^, Juan-Juan Sun^1^, Liang-Ping Xiao^1^, Shiyuan Zhou^1^, Karen C. Bustillo^3^, Long-Hui Lin^1^, Jun Cheng^1^, Jian-Feng Li^1^, Hong-Gang Liao^1,^*, Shi-Gang Sun^1^, Haimei Zheng^2,4,^*

**Affiliations:**

^1^State Key Laboratory for Physical Chemistry of Solid Surfaces, Collaborative Innovation Center of Chemistry for Energy Materials, College of Chemistry and Chemical Engineering, Xiamen University, Xiamen 361005, China.

^2^Materials Science Division, Lawrence Berkeley National Laboratory, Berkeley, CA 94720, USA.

^3^National Center for Electron Microscopy, Molecular Foundry, Lawrence Berkeley National Laboratory, Berkeley, CA 94720, USA.

^4^Department of Material Science and Engineering, University of California, Berkeley, CA 94720, USA.

^#^Present address: National Center for Electron Microscopy, Molecular Foundry, Lawrence Berkeley National Laboratory, Berkeley, CA 94720, USA.

^*^To whom correspondence should be addressed; Email: [hmzheng@lbl.gov](mailto:hmzheng@lbl.gov) (H. Zheng) and [hgliao@xmu.edu.cn](mailto:hgliao@xmu.edu.cn) (H. G. Liao).

**
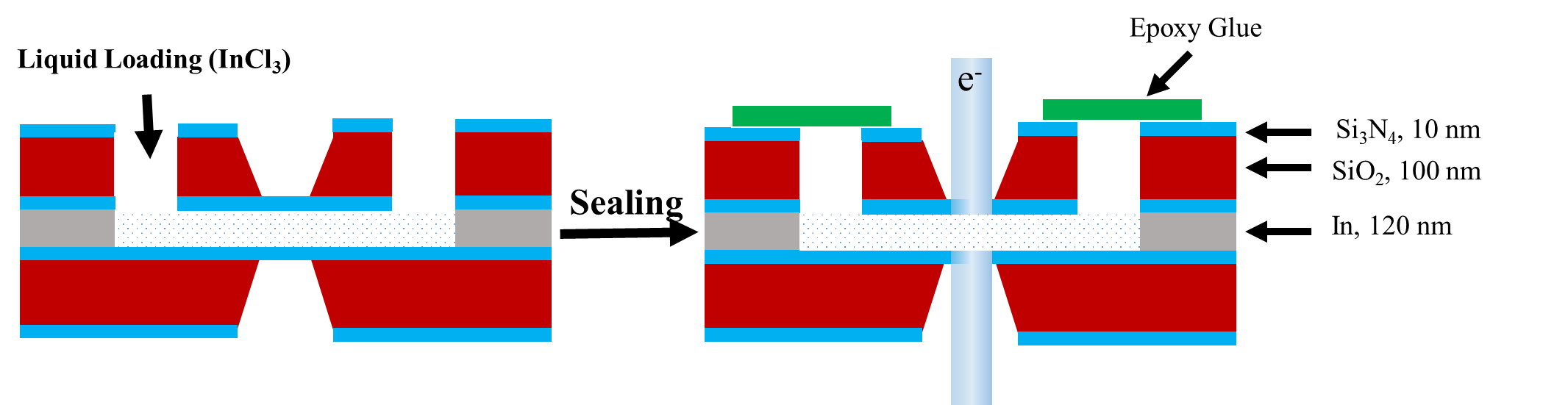
**

**Supplementary Fig. 1** **Illustration of experimental preparation of nanocrystal growth using liquid cell**. InCl_3_ solution was loaded into the liquid cell as the growth solution (see experimental sections for details). In nanocrystals will nucleate and grow using InCl_3_ as a growth solution under electron beam irradiation.


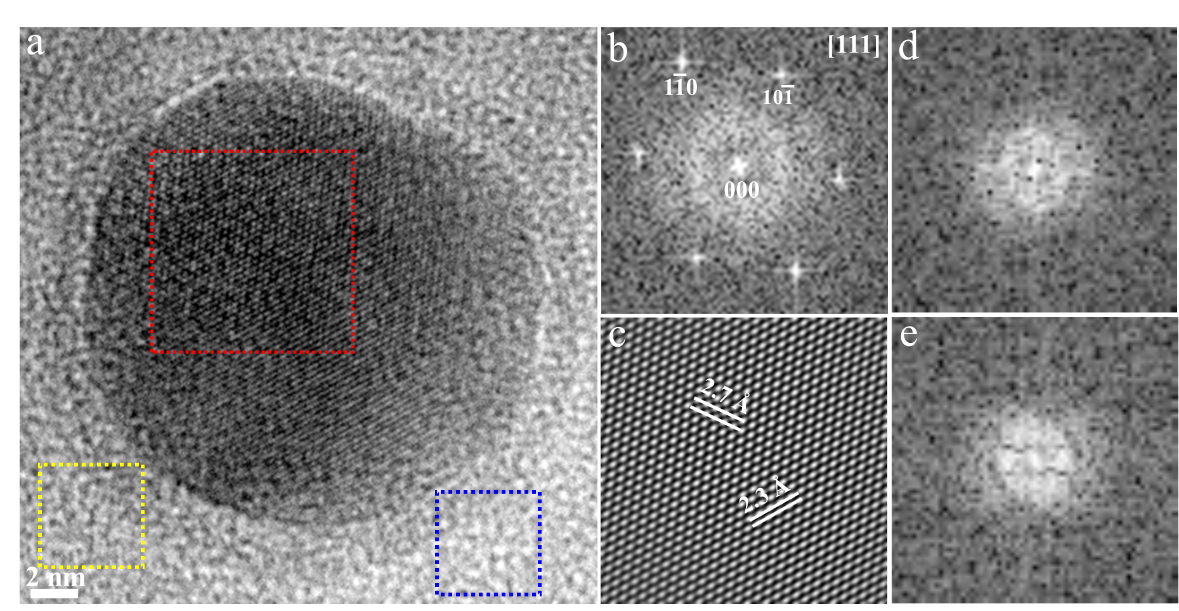


**Supplementary Fig. 2 Structure analysis of core-interphase nanocrystal in the liquid cell. a** High-magnification TEM image of an In nanocrystal in solution. **b** FFT and **c** inverse FFT images from the region by red dashed line in a. **d** FFT of the yellow square area in a. **e** FFT of the blue region in a. The crystalline core and the amorphous phase of the shell are determined.


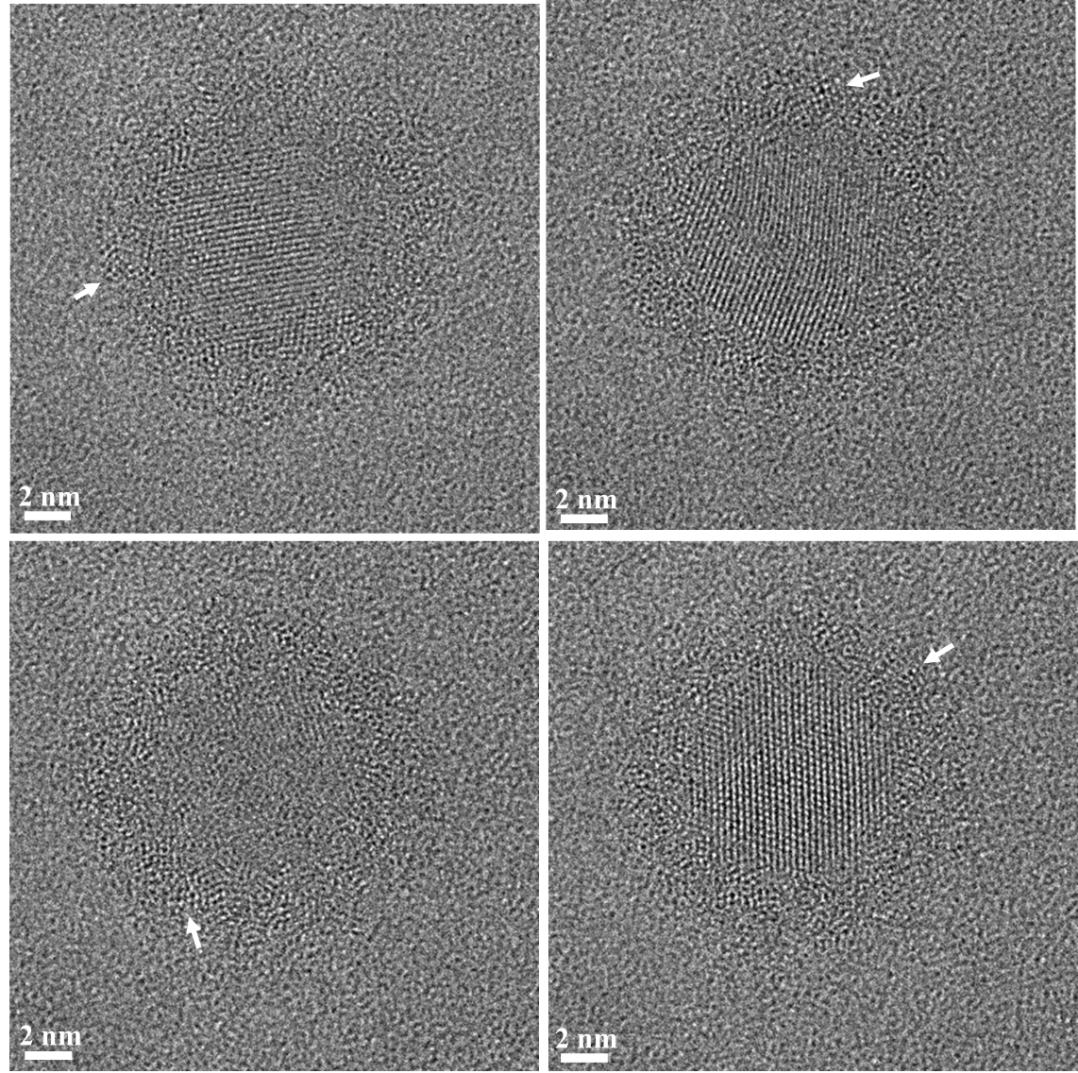


**Supplementary Fig. 3 Representative high-resolution TEM images of In nanocrystals with quasi-liquid phase.** Small In nanoclusters/nanocrystals are indicated by white arrows.


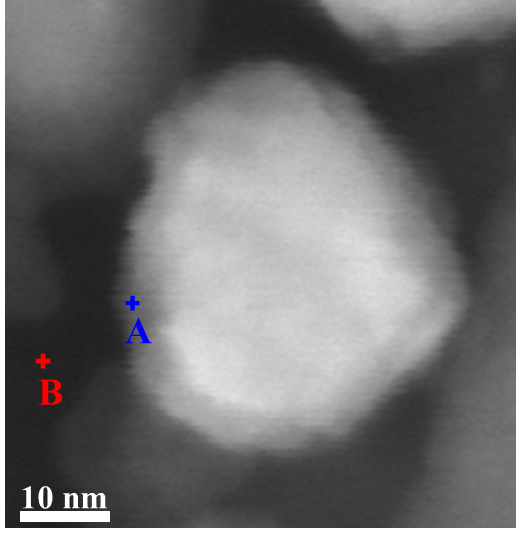


**Supplementary Fig. 4 HAADF-STEM image of In nanocrystal with quasi-liquid phase for EELS.** EELS of quasi-liquid phase and solution are collected from points of A and B, respectively.


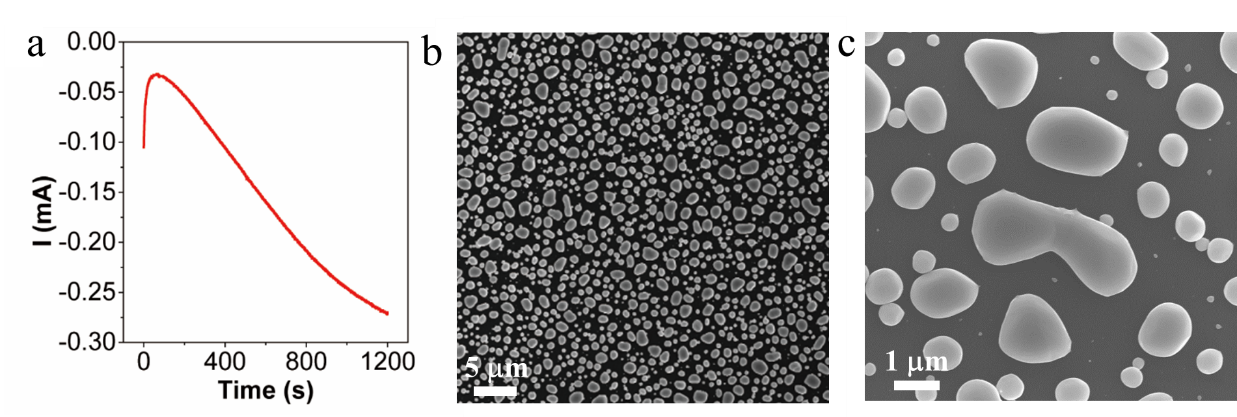


**Supplementary Fig. 5 In nanocrystals synthesized by electrochemical deposition with inert gas protection.** **a** Cyclic voltammetry curve of the electro-deposition of In nanocrystals on a glassy carbon electrode. **b** Low-magnification and **c** high-magnification SEM images of the electro-deposited In nanocrystals.


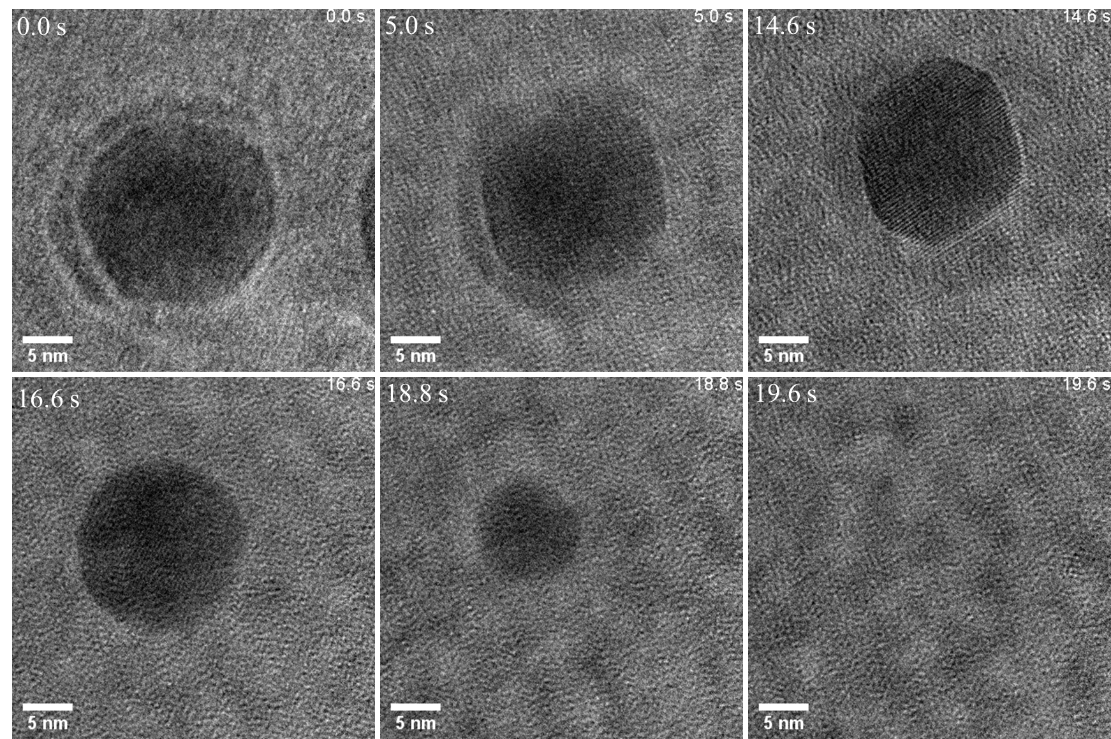


**Supplementary Fig. 6 In-situ observation of the etching of In nanocrystal with quasi-liquid phase.** The quasi-liquid phase disappears when the particle size of In becomes smaller (e.g., the image at 16.6 s).


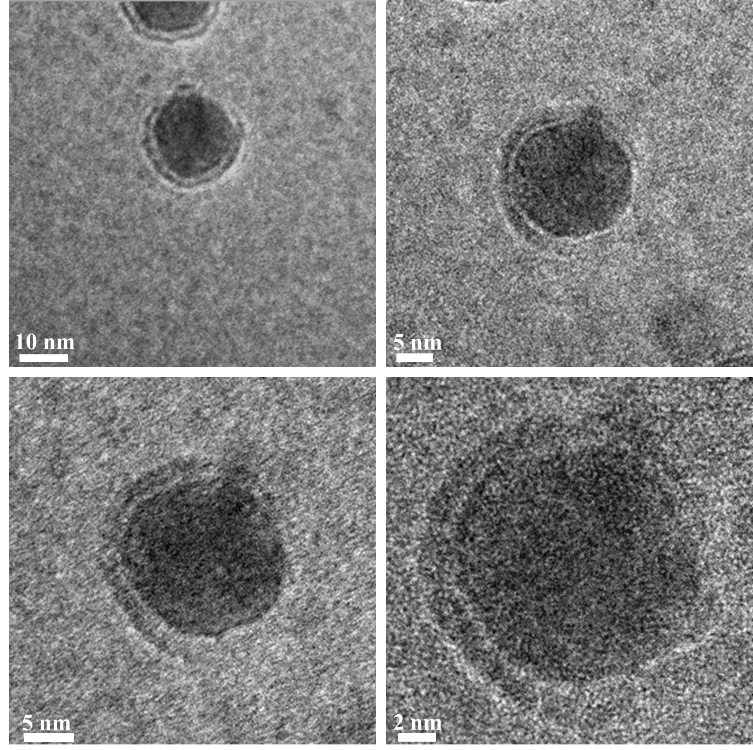


**Supplementary Fig. 7 TEM images of the same core-interphase particle at different magnification.** The images from low to high magnification correspond to the dose rate change from low to high dose accordingly. The dose rates used for these four TEM images are 73.3, 161, 285 and 559 e^-^·Å^-2^·s^-1^, respectively.


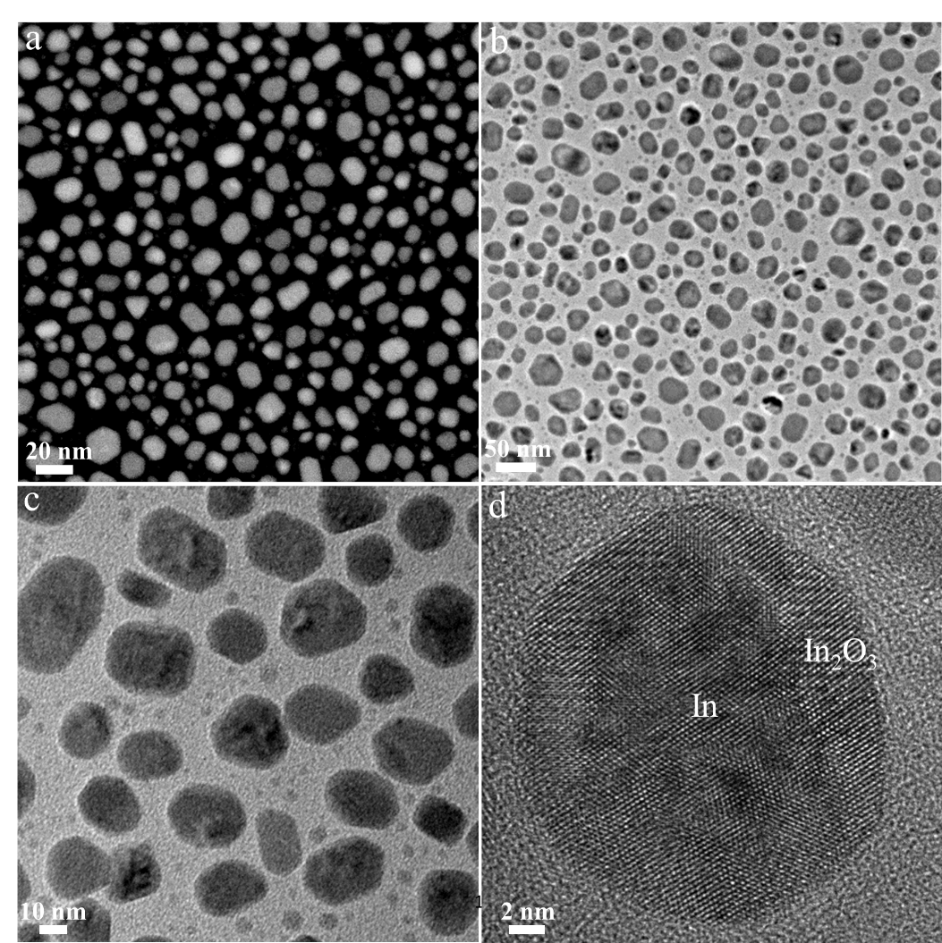


**Supplementary Fig. 8 In@In_2_O_3_ nanocrystals synthesized on the bottom window of a liquid cell by thermal deposition.** **a** HAADF-STEM and **b-c** TEM images of In@In_2_O_3_ nanocrystals synthesized by thermal evaporation. **d** High-magnification TEM image shows In core with oxide shell.


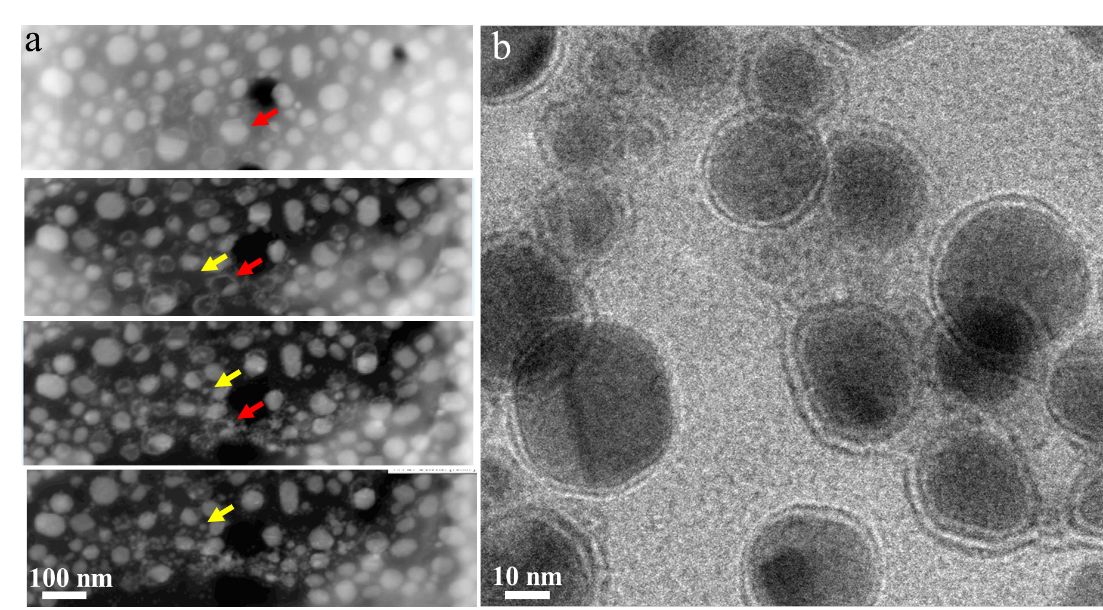


**Supplementary Fig. 9 Sequential STEM images showing dynamic behaviors of In@In_2_O_3_ nanocrystals in water. a** The In core gradually dissolves into the water to form a hollow structure (indicated by the red arrow). Then, some new In nanocrystals will be formed in the neighboring area (indicated by the yellow arrow). **b** New formed nanocrystals display the quasi-liquid phase on the surface.


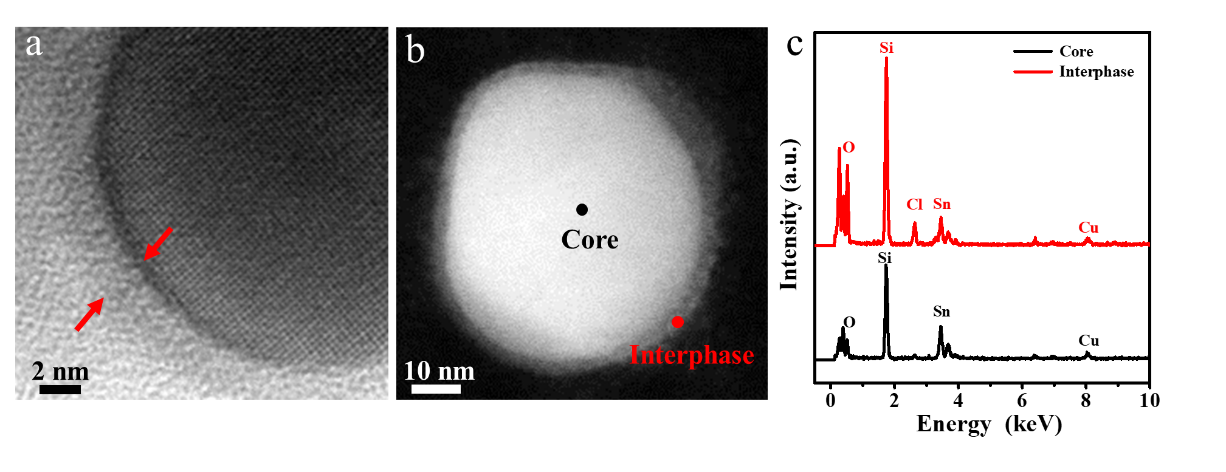


**Supplementary Fig. 10 In-situ observation and structure characterization of the quasi-liquid phase on the surface of Sn nanocrystal in SnCl_4_ solution using liquid-phase TEM.** **a** HRTEM and **b** STEM images of Sn nanocrystal with amorphous interphase in solution. **c** EDS analysis of the core and interphase.


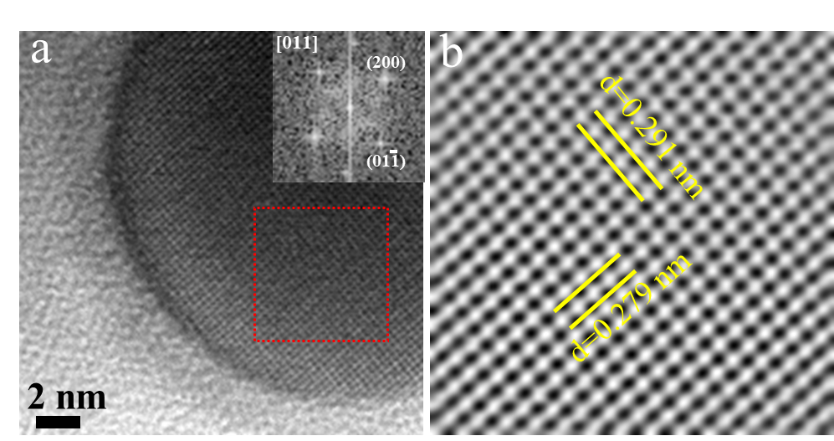


**Supplementary Fig. 11** **Structure determination of Sn core using HRTEM. a** HRTEM image of the Sn core with quasi-liquid phase and corresponding FFT pattern of the selected red square region is shown in the inset. **b** Inverse FFT of the selected red square region.

**
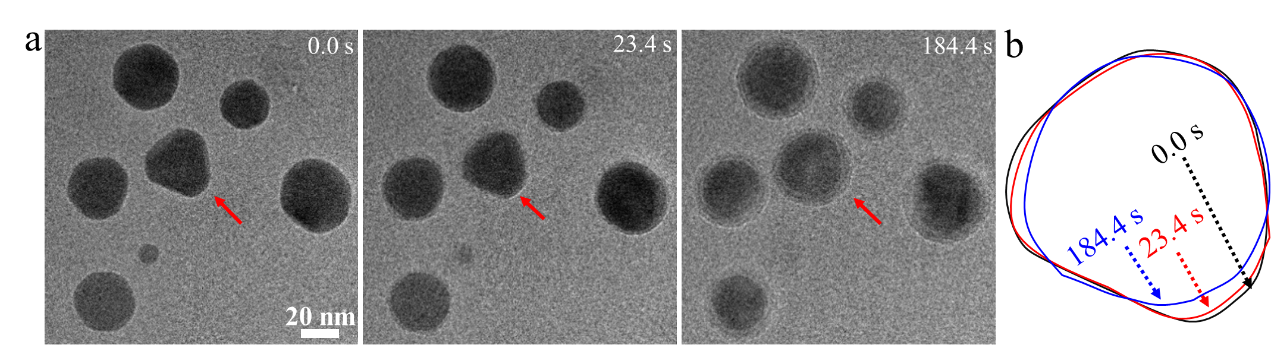
**

**Supplementary Fig. 12** **Formation of the quasi-liquid phase during Sn nanocrystals dissolution.** **a** Sequential TEM images showing the formation of the interphase. **b** The contour of the Sn core (as indicated by the red arrow in **a**) of images in a.


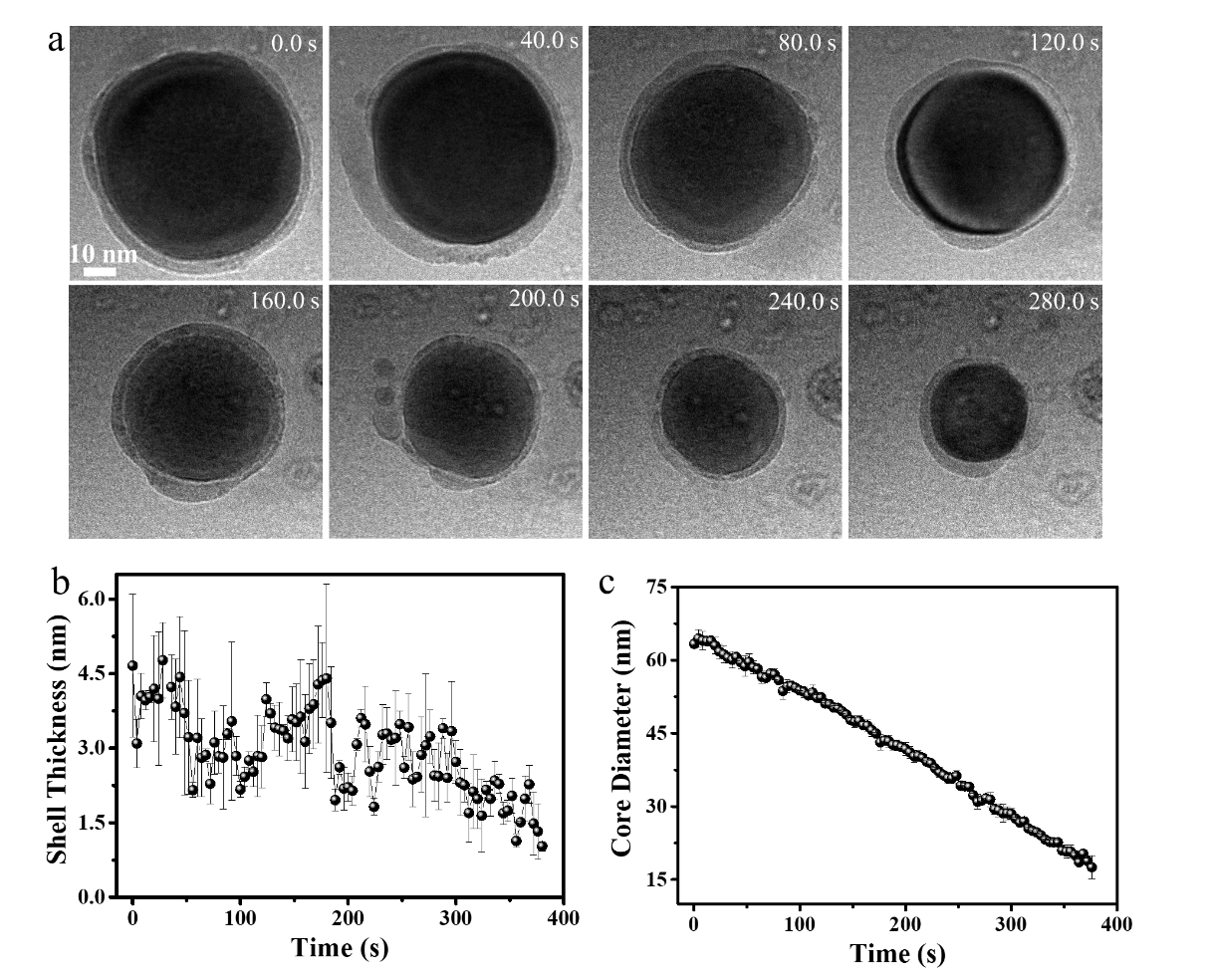


**Supplementary Fig. 13 Mass transport between metal core, quasi-liquid phase and bulk solution. a** Sequential TEM images showing that the core gradually transforms into the interphase and that diffuses into the solution to maintain a certain thickness. **b** Plots of interphase thickness and **c** diameter of metal core as a function of time.


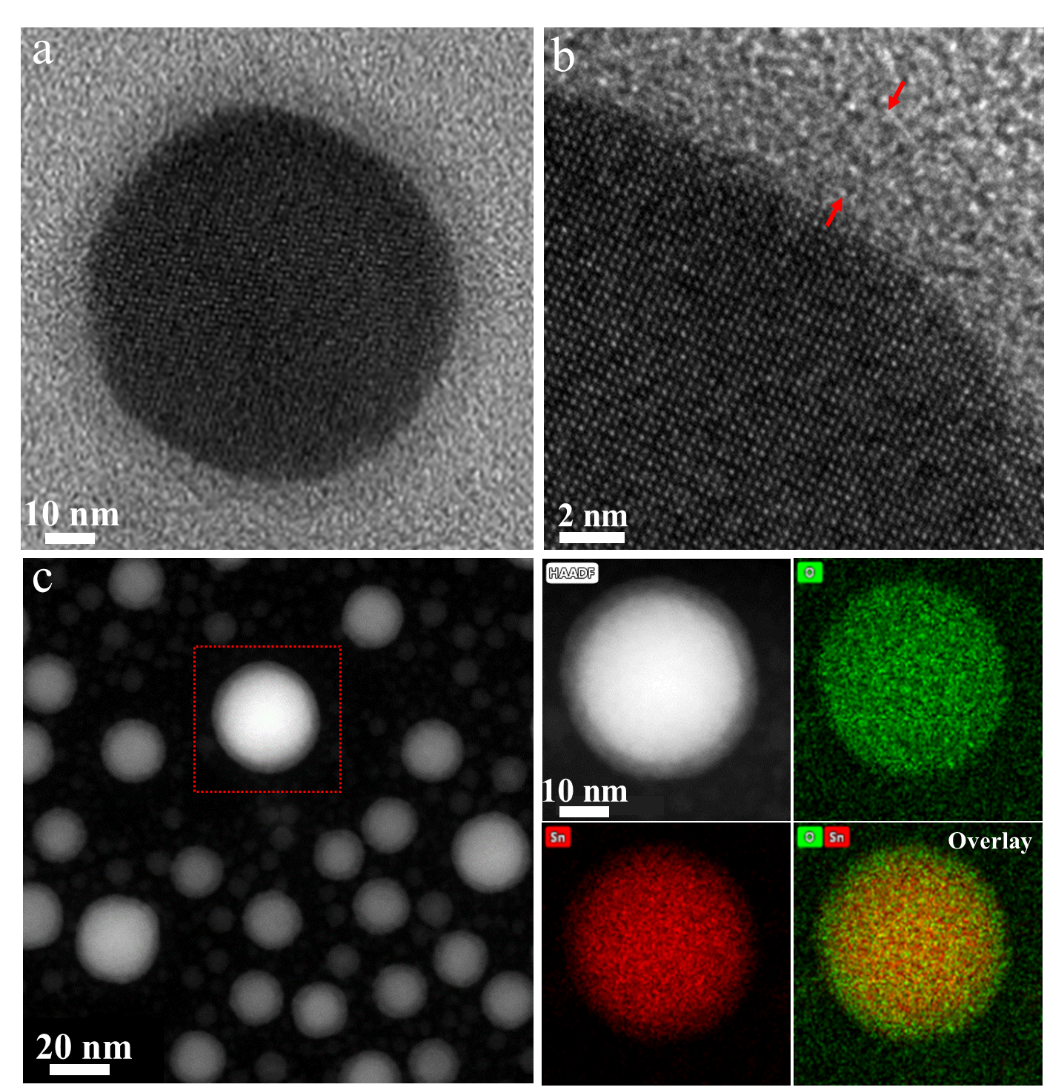


**Supplementary Fig. 14 Structure and composition analysis of Sn@SnO_x_ nanocrystals synthesized by thermal deposition.** **a** Low- and **b** high-magnification TEM images of the Sn-SnO_x_ core-shell structure synthesized by thermal deposition. The amorphous oxide layer is indicated by the red arrow. **c** HAADF-STEM image of Sn-SnO_x_ core-shell structure and corresponding elemental mapping of Sn (green) and O (red).

**
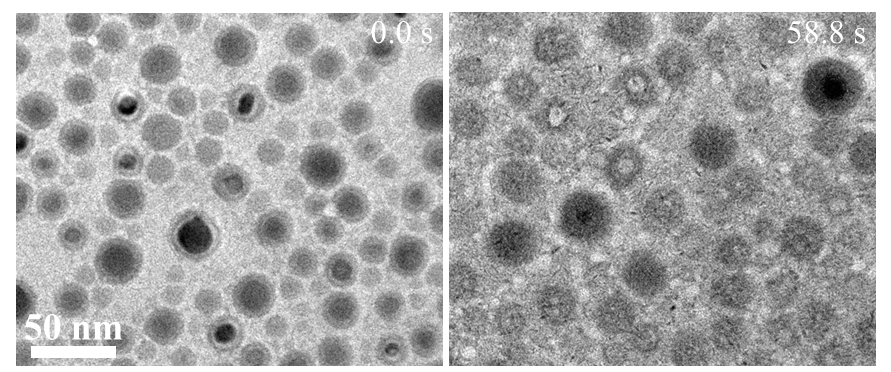
**

**Supplementary Fig. 15 In-situ observation of Sn-SnO_x_ nanocrystals in H_2_O.** The Sn core dissolved in the water to form a hollow structure under beam irradiation from 0.0 to 58.8 s.

**
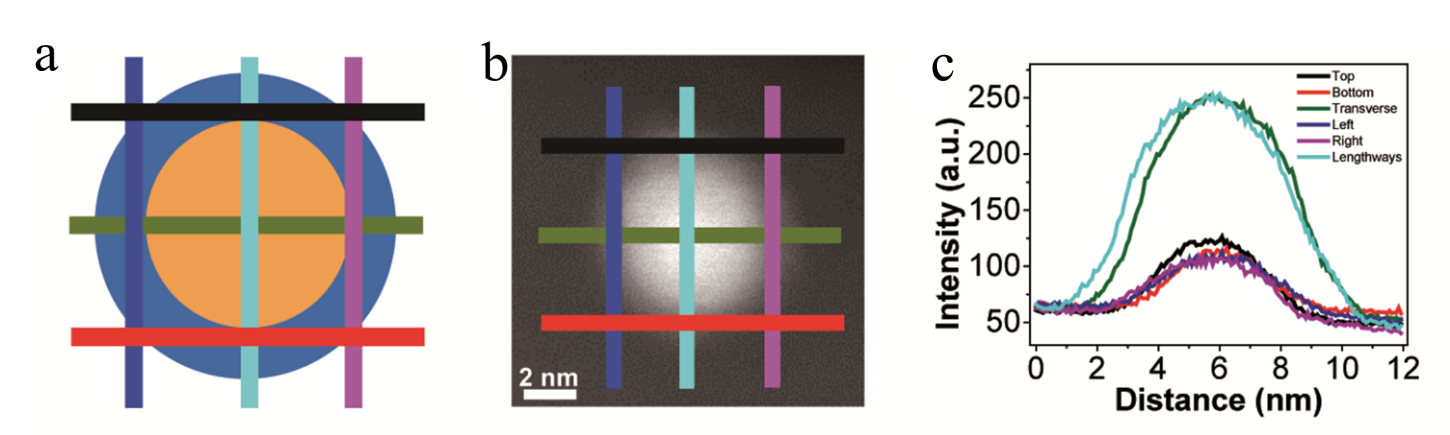
**

**Supplementary Fig. 16 The calculation of atomic concentration of In through the model specification.** **a** The model specification of In core and interphase. **b** HAADF-STEM image of the In nanocrystal in the liquid cell. **c** The mass contrast intensity curves of different directions are marked in a.


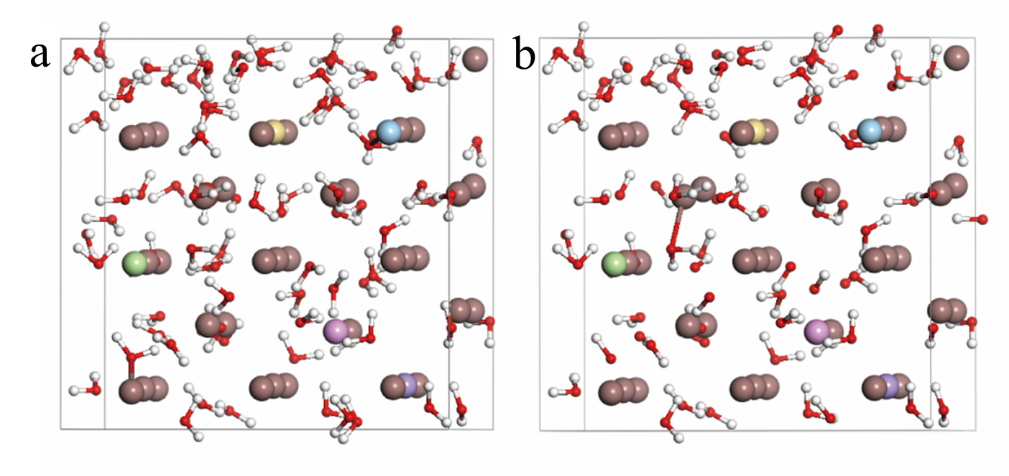


**Supplementary Fig. 17 DFT-MD simulations of the initial structure under the electrically neutral condition and the positively charged condition. a** The initial structure under the neutral condition. A total of 40 In atoms are uniformly distributed in 80 H_2_O molecules. **b** The initial structure under the positively charged condition. The positively charged model is created by removing 40 H from the electrically neutral model. The blue, yellow, purple, green, and pink spheres represent In with different sequence numbers.


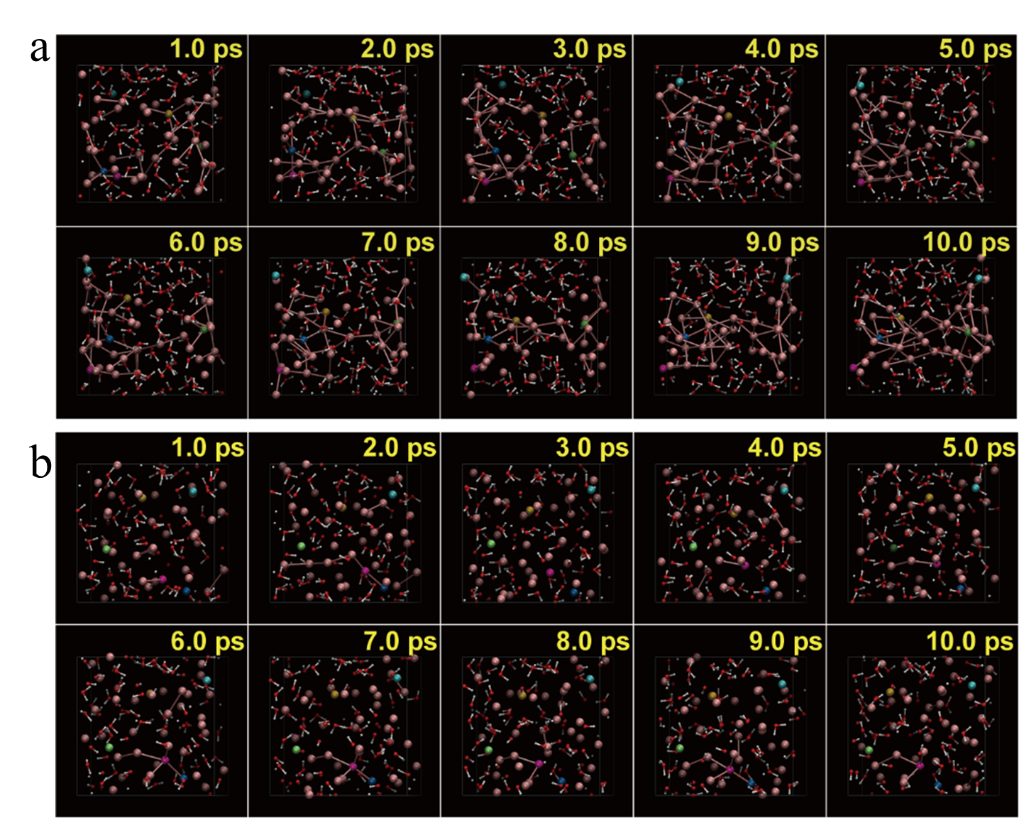


**Supplementary Fig. 18 DFT-MD simulations showing structure evolution under the electrically neutral and the positively charged condition. a** Intermediate structure under the neutral condition and **b** intermediate structure under the positively charged condition.


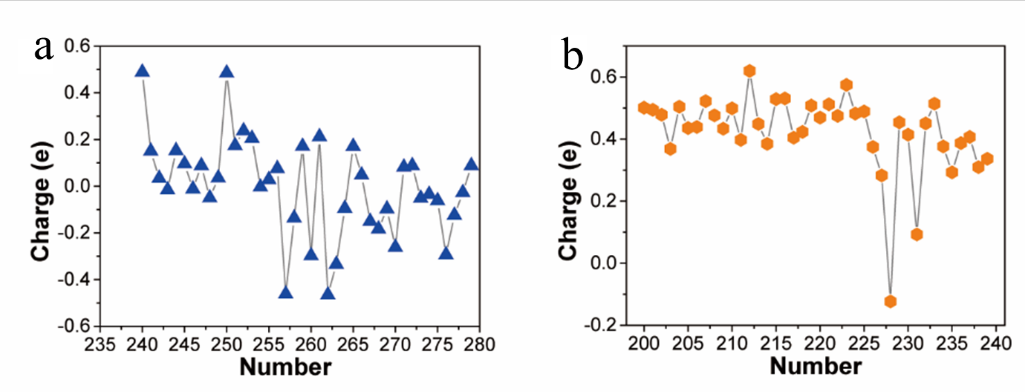


**Supplementary Fig. 19 Charge distribution of 40 In atoms in the final structure from DFT-MD.** **a** The charge of 40 In atoms in the final structure under the electrically neutral condition. **b** The charge of 40 In atoms in the final structure under the positively charged condition.
