# Supplementary material for: Identification of a quasi-liquid phase at solid–liquid interface
Source: Nat Commun. 2022 Jun 23;13:3601. doi: 10.1038/s41467-022-31075-z (PMC9226024; doi:10.1038/s41467-022-31075-z)
Supplement: Supplementary file 3 — Description of Additional Supplementary Files [file 41467_2022_31075_MOESM3_ESM.docx]

Supplementary Movie 1

High-resolution dynamic imaging of In nanocrystals with the quasi-liquid phase in solution. 20 mg/mL InCl_3_. Dose rate: 994 e^-^·Å^-2^·s^-1^.

Supplementary Movie 2

High-resolution dynamic imaging of In nanocrystals with quasi-liquid phase; In nanocrystals in H_2_O. Dose rate: 559 e^-^·Å^-2^·s^-1^.

Supplementary Movie 3

High-resolution dynamic imaging of In nanocrystals with quasi-liquid phase using aberration-corrected TEM. 20 mg/mL InCl_3_. Dose rate: 3750 e^-^·Å^-2^·s^-1^.

Supplementary Movie 4

The etching process of the In core with quasi-liquid phase. 20 mg/mL InCl_3_. Dose rate: 994 e^-^·Å^-2^·s^-1^.

Supplementary Movie 5

Growth of In nanocrystals and formation of quasi-liquid phase. 20 mg/mL InCl_3_. Dose rate: 73.3 e^-^·Å^-2^·s^-1^.

Supplementary Movie 6

The etching process of the In core with quasi-liquid phase. 20 mg/mL InCl_3_. Dose rate: 904 e^-^·Å^-2^·s^-1^.

Supplementary Movie 7

The etching process of the Sn core with quasi-liquid phase. 20 mg/mL SnCl_4_. Dose rate: 285 e^-^·Å^-2^·s^-1^.

Supplementary Movie 8

Nucleation and growth of In nanoclusters from the quasi-liquid phase. 20 mg/mL InCl_3_. Dose rate: 994 e^-^·Å^-2^·s^-1^.

Supplementary Movie 9

Quasi-liquid phase swallowing a small In nanocluster nearby in aqueous solution. 20 mg/mL InCl_3_. Dose rate: 420 e^-^·Å^-2^·s^-1^.

Supplementary Movie 10

The electrically neutral model contains 40 In atoms and 80 H_2_O molecules in a cubic box with a length of 15.31 Å.

Supplementary Movie 11

The “charged” model contains 40 In atoms, 40 H_2_O molecules, and 40 OH molecules in a cubic box with a length of 15.31 Å.
